# Supplementary figures and images for: The recovery of visuospatial neglect with standard treatment: a systematic review and meta-analysis
Source: Stroke. Author manuscript; Available in PMC 2024 Aug 27. (PMC11346719; doi:10.1161/STROKEAHA.124.046760)

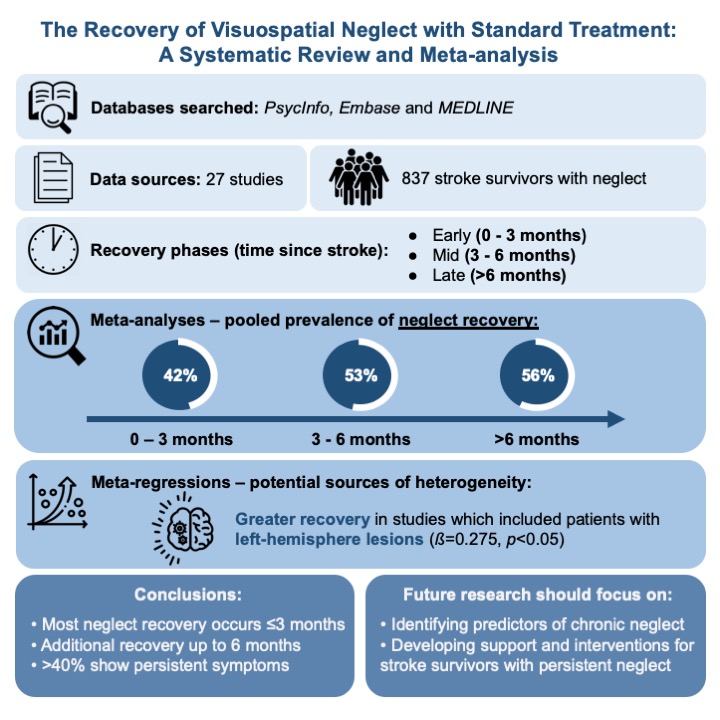

Supplement: Graphical Abstract [file EMS197074-supplement-Graphical_Abstract.jpg]
